# Bilateral large cell calcifying Sertoli cell tumours: A testicular preservation approach in a young male

**DOI:** 10.1016/j.eucr.2023.102628

**Published:** 2023-11-25

**Authors:** Ibrahim A. khalil, Mohamed Hatem, Khaled A. Murshed, Majd Alkabbani, Nagy Younes, Khalid Al Rumaihi

**Affiliations:** a Department of Urology, Hamad Medical Corporation, Doha, Qatar; b Department of Anatomic Pathology, Hamad Medical Corporation, Doha, Qatar

**Keywords:** Large cell calcifying Sertoli cell tumours, Carney complex, Testicular preservation

## Abstract

Testicular Large cell calcifying Sertoli cell tumours (LCCSTs) are extremely rare. The primary challenge in benign LCCSTs, which are typically multifocal and bilateral tumours affecting young males, is to confirm the diagnosis to avoid radical intervention and preserve fertility potential. Patient clinical presentation, laboratory results, diagnostic radiological tests along with confirmatory histopathological studies, are the cornerstones in such cases, nevertheless genetic testing is warranted, as LCCSTs can be part of genetic syndrome such Carney complex. We present a case of bilateral benign LCCSTs in young male managed with testicular preservation approach with characteristic clinical, radiological and histopathological features.

## 1 Introduction

Sertoli cell tumours comprise approximately 1 % of all testicular tumours. Sertoli cell tumours are further categorized as large cell calcifying Sertoli cell tumor (LCCSCT), sclerosing Sertoli cell tumour or Sertoli cell tumour not otherwise specified.^1^ The radiological features of LCCSCT can suggest the diagnosis but a confirmatory histopathologic diagnosis is mandatory to proceed with testicular preservation approach. The traditional approach of radical orchiectomy of such tumours may be an overtreatment of a benign pathology especially in cases of bilateral testicular involvement. In such instances,a testicular preservation approach is advisable to maximize future fertility potential.

We report a case of bilateral multiple LCCSCT with characteristic radiological and histopathological features in a young male, managed with testicular sparing surgery, avoiding radical orchiectomy.

## 2 Case presentation

An 18-year-old gentleman previously healthy with past surgical history of an excision of left lower eyelid benign melanocytic nevus. presented to the emergency department with bilateral scrotal pain for one day. There was no history of trauma and no features of infection. On physical examination, both testicles were nontender and mobile with multiple hard masses. Additionally, he had skin pigmentations and freckling on his back. The laboratory workup was within normal limits. Serum tumour markers including LDH, AFP and BhCG were also normal (Table 1).

**Table 1:** Basic laboratory and tumor markers results.

Test (Units)	Results	Normal reference Range
LDH (U/L)	209	135–225
AFP IU/mL	2	0–6
BhCG (mIU/mL)	<1	0–2
WBC count	5.7 × 10^∧^3/uL	4.0–10.0
Hgb (gm/dL)	13.9	13.0–17.0
Creatinine (umol/L)	69	62–106

**Fig. 1:**
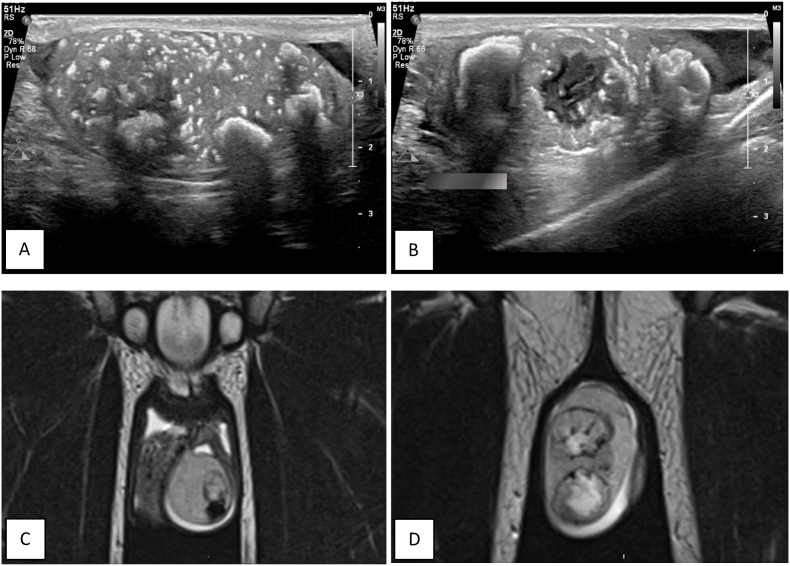
Radiological evaluation, A and B: US showing multiple lobulated heterogenous lesions with extensive calcifications bilaterally, C and D: MRI scan showing multiple bilateral testicular masses with calcifications and cystic changes without extra testicular extension.

Radiological evaluation, Testicular ultrasound (US) showed Extensive calcifications in both testes with multiple lobulated heterogenous lesions (Fig. 1A and B), these findings raised concerns of benign pathology for which a Magnetic Resonance Imaging (MRI) was done and showed six masses on the right testis and three on the left testis, with calcifications and cystic change and no signs extra testicular invasion (Fig. 1C and D). PET-CT and staging CT scans were unremarkable for metastatic disease.

Surgical intervention, after sperm cryopreservation, patient underwent right inguinal excisional testicular biopsy with excision of four well-circumscribed lesions, each less than one cm, surrounded by healthy testicular tissue which was preserved.

**Fig. 2:**
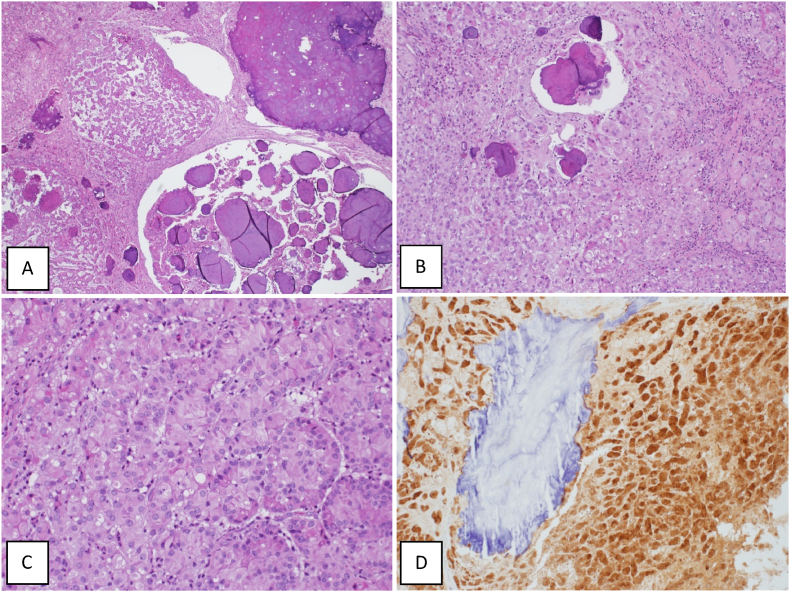
Histopathological diagnosis: A: Photomicrograph reveals a tumor with multinodular growth pattern and prominent large calcifications (Hematoxylin & Eosin stain, 40x). B: The tumor cells are arranged in cords and nests in a stroma rich in inflammatory cells with calcifications (Hematoxylin & Eosin stain, 100x). C: The tumor is composed of Sertoli cells that are polygonal and have eosinophilic cytoplasm with rounded nuclei and prominent nucleoli (Hematoxylin & Eosin stain, 200x). D: The tumours cells are diffusely and strongly reactive for calretinin antibody (Immunohistochemistry, 200x).

Histopathology, sections show a well-circumscribed testicular tumour composed of lobules of cells arranged in solid sheets and cords. The tumour cells are epithelioid and have a round nucleus, prominent nucleoli with abundant eosinophilic cytoplasm. There are multiple large, laminated calcifications, there is no evidence of marked pleomorphism, necrosis, or atypical mitosis (Fig. 2A–C).

By immunohistochemistry, the tumour cells are strongly and diffusely positive for calretinin, inhibin and S100, with focal positivity for Melan-A and cytokeratin AE1/AE3. However, the tumour cells are negative for EMA. The proliferative index Ki-67 is very low (less than 2 %) (Fig. 2D).

A final diagnosis of large cell calcifying Sertoli cell tumour was made. Genetic testing is positive for a pathogenic mutation c.491_492del in the PRKAR1A gene-confirming Carney Complex. Patient was kept on follow up for any signs of extra testicular extension or involvement of other systems as part of carney complex. The case is followed annually by cardiology, endocrine, dermatology, and orthopedic team along with follow up with urology for annual testicular ultrasound to detect the development of any new lesions.

## 3 Discussion

LCCSCTs are rare testicular tumours with a frequency of 0.4–1.5 %. Benign LCCSTs are multifocal and bilateral tumours with a mean age of presentation of 17 years, compared to the malignant LCCSTs are typically unifocal and unilateral disease that occur in older patients with mean age of 39 years.^1^ Although most of the cases are sporadic (60 %) but is also linked to multiple neoplastic syndromes such as Peutz–Jeghers syndrome and Carney complex^2^ as seen in our case with cutaneous manifestations of cutaneous myxomas and LCCST. The main dilemma in Benign LCCSTs is confirming the diagnosis to avoid radical intervention, especially in cases of bilateral disease, to preserve fertility potential. As in our case, an 18-year-old male with bilateral tumours, the endocrine and fertility functions of the testis were of major concerns.

Radiological evaluation plays a key role in diagnosing LCCSTs. For instance, ultrasound can demonstrate bilateral testicular enlargement, with or without microcalcifications, increased vascular flow in proximity to the calcified area on colour doppler ultrasound, along with the absence of soft or cystic tissues. These ultrasonographic features rises the suspicion of LCCSCTs. Nevertheless, the characteristic ‘Christmas tree-like’ appearance of multiple lesions in syndromic LCCSCTs are almost pathognomonic. LCCSCTs can also be seen as a round, smooth, and hyperechogenic area, or multiple hyperechogenic spots.^1^^,^^2^ The main role of MRI is to exclude other causes like lymphoma, as LCCSCTs have no specific appearance on MRI.^3^ In our case the main suggestive feature of LCCSCT was the testicular US features of extensive bilateral calcifications, the MRI only ruled out other cases with no added value in the radiological diagnosis.

LCCSCT has peculiar morphology. The tumour shows nodular growth pattern composed of nests and trabeculae of pale to eosinophilic epithelioid cells interspersed with hypocellular myxoid stroma rich in neutrophils. Potentially malignant tumours are characterized by the presence of ≥1 adverse pathologic features; size>4 cm, extra testicular growth, necrosis, marked nuclear atypia, vascular invasion, and >3 mitotic figures/10 HPFs.^4^ By immunochemistry, the tumour shows immunoreactivity for inhibin, vimentin, calretinin, and S100 protein.^4^ It has been recently found that PRKAR1A loss is highly sensitive and specific for the diagnosis of LCCSCT particularly in sporadic cases and those that are associated with Carney complex.^5^ The differentiation between malignant and benign cases requires clinical, radiological and histopathological features with immunochemical tests, as detailed above.

We report a rare case of bilateral benign LCCSCT with typical features on radiological and histopathological evaluation, treated with excisional biopsy and preservation of healthy testicular tissue.

## 4 Conclusion

LCCSCTs are rare testicular tumours; diagnosis requires an interaction between clinical, radiological, and histopathological features. Given the benign nature of LCCSCTs, it is advisable to consider a testicular-preserving approach once malignant masses are ruled out."

## CRediT authorship contribution statement

**Ibrahim A. khalil:** Conceptualization, Data curation, Methodology, Validation, Visualization, Writing – original draft, Writing – review & editing, Investigation. **Mohamed Hatem:** Conceptualization, Writing – original draft. **Khaled A. Murshed:** Data curation, Investigation, Writing – original draft. **Majd Alkabbani:** Validation, Writing – review & editing. **Nagy Younes:** Conceptualization, Supervision. **Khalid Al Rumaihi:** Resources, Supervision, Validation.

## Declaration of competing interest

The authors declare that they have no financial or non-financial conflicts of interest related to the subject matter or materials discussed in the manuscript.

## Abbreviations

LCCSCT: large cell calcifying Sertoli cell tumour
LDH: Lactate dehydrogenase
AFP: Alpha fetoprotein
BhCG: Beta human chorionic gonadotropin
US: ultrasound
MRI: Magnetic Resonance Imaging
PET: positron emission test
CT: computed tomography
HPF: High power field
